# Higher C-reactive protein/albumin ratio is a potential marker for predicting amputation in patients with diabetic foot infection

**DOI:** 10.20945/2359-4292-2024-0397

**Published:** 2025-06-27

**Authors:** Li Zhang, Xufeng Gao, Meifang He, Wenyan Wang, Yuebin Zhao

**Affiliations:** 1 Department of Endocrinology, Taiyuan City Central Hospital, Taiyuan, Shanxi, China; 2 Department of General Surgery, Children’s Hospital of Shanxi, Taiyuan, Shanxi, China

**Keywords:** Diabetic foot infection, C-reactive protein, albumin, amputation

## Abstract

**Objective:**

Non-traumatic amputation resulting from diabetic foot infection (DFI) poses significant clinical and public health concerns. The C-reactive protein (CRP)/albumin ratio represents a combination of the infection level and nutritional status. This study investigated the relationship between the CRP/albumin ratio and amputation in patients with diabetic foot infections.

**Subjects and methods:**

Patients with a DFI of Wagner grade. 3 diagnosed between January 2020 and September 2023 were retrospectively analyzed. The association between the CRP/albumin ratio and amputation was explored using multivariable logistic regression modeling. Stratified analyses were also performed to ensure the reliability of the findings.

**Results:**

Of 301 enrolled patients, 226 underwent amputation and 75 did not. The amputation rate increased with a greater CRP/albumin ratio in the non-adjusted, minimally adjusted, and fully adjusted models, regardless of whether the CRP/albumin ratio was regarded as a categorical or continuous variable.

**Conclusion:**

An increased CRP/albumin ratio was associated with a greater risk of amputation in individuals with DFI.

## INTRODUCTION

Diabetic foot ulcers (DFUs) are a combination of foot deformities, peripheral arterial disease, neuropathy, and infections (^1^). Approximately 50%-60% of patients with a DFU develop diabetes-related foot infections (DFIs) (^2^). Foot ulcer wounds that are difficult to heal for a long duration can seriously affect the quality of life of diabetic patients (^3^) and are closely related to a high incidence of lower limb amputation and increased mortality (^4^). Approximately 20% of moderate-to-severe DFIs result in amputation of the lower extremities, and the worldwide 5-year mortality rate for patients with severe amputations exceeds 70% (^2^,^5^). In addition, the worldwide burden of DFIs has been growing annually (^6^,^7^), imposing a huge economic and social burden on families and society.

Currently, the clinical identification of DFI is based on the signs or symptoms of local or systemic inflammation (^8^). The International Working Group on the Diabetic Foot (IWGDF) recommends defining severe DFI as any foot infection accompanied by the systemic inflammatory response syndrome (^8^). C-reactive protein (CRP) is an early stage response substance that has been widely accepted as a sensitive inflammatory serum biomarker. The CRP level is higher among infected patients than controls (^9^) and, therefore, has been implicated as a potential marker of many diseases. However, the CRP level is not a specific indicator of a single disease (^10^). Recent research suggests that the CRP/albumin ratio may be a novel marker of infection severity (^11^). This ratio shows promising potential as a predictive marker for some inflammation-related diseases such as acute pancreatitis (^12^,^13^), cancer (^14^-^18^), stroke (^19^,^20^), cardiovascular diseases (^21^-^23^), and COVID-19 (^24^-^26^). However, only one study has investigated the connection between the CRP/albumin ratio and amputation risk in patients with a DFI (^27^), and no data from China have been reported.

Therefore, this study evaluated the correlation between the CRP/albumin ratio and amputation risk in patients with DFI in a Chinese tertiary care hospital. We anticipate that our results will help clinicians predict the likelihood of amputation at the patient’s initial visit and take timely and effective measures to avoid or minimize amputation.

## SUBJECTS AND METHODS

This retrospective study was conducted in accordance with the Declaration of Helsinki and approved by the Ethics Review Committee of our hospital (ID: 2024001). The requirement for informed consent was waived because the patient data were anonymized. Consecutive data of patients diagnosed with DFI at our hospital between January 2020 and September 2023 were retrospectively reviewed. DFI was diagnosed based on IWGDF guidelines (^8^), and the severity of DFU was graded according to Wagner’s classification (^28^). The inclusion criteria were 1) diagnosis of Wagner grade ≥ 3 DFI, 2) age ≥ 18 years old, and 3) complete clinical data. The exclusion criteria were 1) DFI with Wagner grade 0, 1, or 2; 2) complications with other inflammatory diseases such as Buerger’s disease, inflammatory bowel disease, rheumatic diseases, or cancer; 3) multiple hospitalizations; and 4) missing data on CRP or albumin levels.

### Data collection

The following data were obtained from our hospital information system: 1) baseline characteristics including sex, age, body mass index (BMI), systolic blood pressure, diastolic blood pressure, smoking status, and complications (hypertension, coronary heart disease, and cerebrovascular disease) and 2) laboratory test results including red blood cell (RBC), white blood cell (WBC), and platelet counts; neutrophil percentage; and hemoglobin, fasting glucose, glycosylated hemoglobin (HbA1c), CRP (mg/L), and albumin (g/L) levels. Laboratory tests were performed on the morning of admission. Smoking status was categorized as current, former, or never smoker. The CRP/albumin ratio was calculated as the ratio of CRP level to albumin level.

Amputations were categorized as minor or major, defined as amputation with the ankle joint left intact and complete wound healing or amputation above the ankle joint, respectively (^29^). DFI without systemic manifestations, involving only the skin or subcutaneous tissue (no deeper tissue), or any erythema that did not extend > 2 cm was regarded as a mild infection. DFI-affected tissues deeper than the skin and subcutaneous tissues with erythema extending > 2 cm without systemic manifestations were classified as moderately affected. DFI was categorized as severe when two or more systemic symptoms were present, such as body temperature > 38 °C or < 36 °C, heart rate > 90 beats/min, respiratory rate > 20 breaths/min, PaCO_2_ < 4.3 kPa (32 mmHg), WBC count > 12,000 or < 4,000 cells/mm^3^, or immature (banded) leukocytes > 10%.

### Statistical analysis

The CRP/albumin ratio was regarded as a categorical variable; patients were categorized based on the tertiles of the CRP/albumin ratio, and patient characteristics were analyzed according to tertiles. Categorical variables are presented as numbers and percentages. The normal distribution of continuous data was initially examined using the Kolmogorov-Smirnov test, and normally distributed data are presented as the mean ± standard deviation and non-normally distributed data as the median and interquartile range. The Chi-square test, Student’s t-test, or Mann-Whitney U test was used to compare categorical variables, normally distributed continuous variables, and non-normally distributed continuous variables, respectively.

Multivariable logistic regression analyses were performed to evaluate the independent association between the CRP/albumin ratio and amputation in patients with DFI. First, analyses were performed without adjustments. The minimally adjusted model was adjusted for age and sex. The fully adjusted model was adjusted for age, sex, smoking status, hypertension, coronary heart disease, cerebrovascular disease, mean arterial pressure (MAP), fasting glucose levels, and HbA1c levels. Subgroup analyses were stratified according to relevant covariates. The diagnostic value of related factors was predicted using receiver operating characteristic (ROC) curves.

All analyses were performed using R statistical software (version 4.2.2; The R Foundation, Indianapolis, IN, USA) (^30^) and Free Statistics Analysis Platform version 1.9. Two-tailed P values < 0.05 were regarded as statistically significant.

## RESULTS

### Baseline characteristics

**Figure 1:**
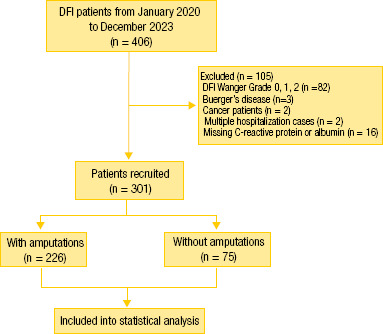
Flowchart of participant enrollment.

Initially, 406 patients were eligible to participate. After excluding patients with Wagner grade 0, 1, or 2; patients with complications such as another inflammatory disease or cancer; and patients with incomplete data, 301 patients, including 208 men and 93 women, were ultimately enrolled and evaluated (Figure 1). Table 1 shows the general characteristics of the participants according to CRP/albumin ratio tertiles. Patients were aged 65.2 ± 12.0 years with an average interval since diabetes diagnosis of 15.4 ± 9.5 years. Sex, age, BMI, MAP, smoking status, diabetes duration, DFI microbial community, coronary artery disease, and cerebral infarction were comparable among the tertiles (P > 0.05). However, individuals with a higher CRP/albumin ratio tended to have a more severe DFI, a higher incidence of hypertension, and higher WBC and platelet counts, neutrophil percentage, and fasting glucose, HbA1c, and CRP levels but lower RBC, hemoglobin, and albumin levels (P < 0.05). The IWGDF DFI grading revealed that 235 individuals (78.1%) had mild and moderate infections, whereas 66 (21.9%) had severe infections. The patients were further divided into mild and moderate infection (n = 235) and severe infection (n = 66) groups. Statistical analysis demonstrated that patients with severe infections had a higher CRP/albumin ratio than those with mild or moderate infections (P < 0.001) (Table S1).

**Table 1:** Baseline characteristics of the study participants

Variables	CRP to albumin ratio				P value
	Total	Triples 1 (≤1.239)	Triples 2 (1.239-3.841)	Triples 3 (≥3.841)	
**Baseline characteristics**					
Sex, n (%)					0.88
Male	208 (69.1)	71 (71)	68 (68)	69 (68.3)	
Female	93 (30.9)	29 (29)	32 (32)	32 (31.7)	
Age, years, mean ± SD	65.2 ± 12.0	66.8 ± 11.7	65.5 ± 11.5	63.3 ± 12.6	0.113
BMI, kg/m^2^, mean ± SD	23.2 ± 3.5	23.6 ± 3.1	22.9 ± 3.9	23.1 ± 3.5	0.34
MAP, mmHg, mean ± SD	59.0 ± 16.9	59.6 ± 18.3	58.6 ± 15.2	58.8 ± 17.1	0.916
Smoking status, n (%)					0.639
Current	60 (19.9)	20 (20)	19 (19)	21 (20.8)	
Never	107 (35.5)	41 (41)	32 (32)	34 (33.7)	
Former	134 (44.5)	39 (39)	49 (49)	46 (45.5)	
Diabetes duration, years, mean ± SD	15.4 ± 9.5	14.8 ± 8.7	15.6 ± 9.1	15.7 ± 10.5	0.777
**DFI severity**					
DFI microbial community, n (%)					0.97
G+ bacterial	100 (34.2)	30 (31.9)	34 (34.3)	36 (36.4)	
G- bacterial	74 (25.3)	25 (26.6)	24 (24.2)	25 (25.3)	
Mixed infection	118 (40.4)	39 (41.5)	41 (41.4)	38 (38.4)	
IWGDF classification, n (%)					<0.001
Mild and moderate	235 (78.1)	98 (98)	82 (82)	55 (54.5)	
Severe infection	66 (21.9)	2 (2)	18 (18)	46 (45.5)	
**Comorbidities**					
Hypertension, n (%)					0.026
Yes	153 (50.8)	43 (43)	48 (48)	62 (61.4)	
No	148 (49.2)	57 (57)	52 (52)	39 (38.6)	
CAD, n (%)					0.319
Yes	214 (71.1)	71 (71)	76 (76)	67 (66.3)	
No	87 (28.9)	29 (29)	24 (24)	34 (33.7)	
Cerebrovascular disease, n (%)					0.38
Yes	205 (68.6)	70 (70.7)	71 (71.7)	64 (63.4)	
No	94 (31.4)	29 (29.3)	28 (28.3)	37 (36.6)	
**Laboratory examination**					
FBG, mmol/L, mean ± SD	10.4 ± 4.9	9.2 ± 4.4	10.4 ± 4.7	11.7 ± 5.3	<0.001
HbA1c, %, mean ± SD	9.0 ± 2.3	8.5 ± 2.4	8.9 ± 2.1	9.5 ± 2.3	0.027
RBC, ×10^12^/L, mean ± SD	3.8 ± 0.7	4.0 ± 0.8	3.8 ± 0.6	3.7 ± 0.7	0.004
Hemoglobin, g/L, mean ± SD	112.8 ± 22.7	120.5 ± 23.5	111.9 ± 20.5	106.1 ± 22.0	<0.001
WBC, ×10^9^/L, mean ± SD	11.6 ± 5.8	8.1 ± 2.7	10.5 ± 3.9	16.1 ± 6.6	<0.001
Neutrophil, %, mean ± SD	78.5 ± 10.4	70.8 ± 10.2	78.7 ± 7.2	86.0 ± 7.5	<0.001
Platelet, ×10^9^/L, mean ± SD	296.4 ± 115.9	258.2 ± 87.7	309.8 ± 120.4	320.9 ± 126.7	<0.001
CRP, mg/L median (IQR)	73.5 (28.2, 136.7)	14.8 (9.3, 27.3)	73.7 (61.3, 91.0)	169.8 (136.7, 218.2)	<0.001
Albumin, g/L, mean ± SD	30.8 ± 6.0	35.0 ± 4.7	30.8 ± 4.2	26.5 ± 5.6	<0.001

Note – Abbreviations: BMI, body mass index; CAD, coronary heart disease; CRP, C-reactive protein; DFI, diabetic foot infections; FBG, fasting blood glucose; G+, Gram-positive bacterial; G-, Gram-negative bacterial; HbA1c, glycosylated hemoglobin; IQR, interquartile range; IWGDF, International Working Group on the Diabetic Foot; MAP, mean arterial pressure; RBC, red blood cell counts; SD, standard deviation; WBC, white blood cell count.

### Relationship between the CRP/albumin ratio and amputation

**Figure 2:**
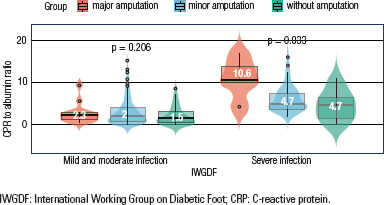
Distribution of CRP/albumin ratio in patients with amputation by severity of diabetic foot infection.

Of 301 patients, 226 (75.1 %) underwent amputation, including 18 major amputations (8.0%) and 208 minor amputations (92.0%). Of the 66 patients with severe infections, 54 (81.8%) underwent amputation. The CRP/albumin ratios of patients who underwent major amputation, minor amputation, and no amputation in the mild and moderate infection group were not significantly different, whereas those who underwent major amputation had the highest CRP/albumin ratios among patients with severe infection (P < 0.05) (Figure 2).

Univariate analysis suggested that MAP, HbA1c, RBC, hemoglobin, WBC, neutrophil percentage, and CRP/albumin ratio were significantly associated with amputation rate (P < 0.05, Table 2). Furthermore, the association between the CRP/albumin ratio and amputation rate was detected using multivariable logistic proportional hazard regression analysis. As shown in Table 3, when the CRP/albumin ratio was incorporated as a continuous variable in the analysis, the amputation rate rose along CRP/albumin ratios in the non-adjusted model (odds ratio [OR]: 1.13, 95% confidence interval [CI]: 1.03-1.25; P = 0.012] (crude model), minimally adjusted model (OR: 1.14, 95% CI: 1.03-1.25; P = 0.008), and fully adjusted model (OR: 1.19, 95% CI: 1.07-1.34; P = 0.002). As a categorical variable, a CRP/albumin ratio ≥ 3.841 indicated a significant association with the amputation rate in the crude model (OR: 2.03, 95% CI: 1.06-3.9; P = 0.033), minimally adjusted model (OR: 2.16, 95% CI: 1.11-4.18; P = 0.023), and fully adjusted model (OR: 2.73, 95% CI: 1.25-5.98; P = 0.012).

**Table 2:** Logistic univariate analysis of the relationship between each indicator and amputation

Variable	OR (95% CI)	P
Gender	1.2 (0.68~2.14)	0.531
Age	1.01 (0.99~1.04)	0.249
BMI	0.93 (0.86~1.01)	0.076
MAP	1.02 (1~1.04)	0.015
Never Smoking	0.45 (0.2~1.03)	0.06
Former Smoking	0.48 (0.21~1.07)	0.074
Diabetes duration (years)	1.03 (1~1.06)	0.095
G+ bacterial	0.82 (0.42~1.59)	0.557
G- bacterial	1.53 (0.81~2.88)	0.19
Severe infection	1.65 (0.83~3.28)	0.155
Hypertension	1.23 (0.73~2.07)	0.443
CAD	1.39 (0.76~2.54)	0.281
Cerebral.infarction1	1.24 (0.7~2.21)	0.459
FBG	1.02 (0.96~1.08)	0.468
HbA1c	0.87 (0.77~0.98)	0.017
RBC	0.64 (0.44~0.94)	0.021
Hemoglobin	0.98 (0.97~0.99)	0.004
WBC	1.06 (1~1.11)	0.045
Neutrophil	1.03 (1~1.05)	0.026
Platelet	1 (1~1)	0.191
CRP	1.01 (1~1.01)	0.008
Albumin	0.96 (0.92~1)	0.075
CRP to albumin ratio	1.13 (1.03~1.25)	0.012

Note – Abbreviations: BMI, body mass index; CAD, coronary heart disease; CRP, C-reactive protein; G+, Gram-positive bacterial; G-, Gram-negative bacterial; HbA1c, glycosylated hemoglobin; MAP, mean arterial pressure; RBC, red blood cell counts; WBC, white blood cell count.

**Table 3:** Associations between CRP to albumin ratio and DFI amputations in the multiple regression model

Variable	Crude model, N = 301	P value	Minimally adjusted model N = 301	P value	Fully adjusted model N = 301	P value
CRP to albumin ratio	1.13 (1.03~1.25)	0.012	1.14 (1.03~1.25)	0.008	1.19 (1.07~1.34)	0.002
CRP to albumin ratio						
≤1.239	Reference		Reference		Reference	
1.239-3.841	1.49 (0.8~2.78)	0.209	1.52 (0.81~2.84)	0.191	1.37 (0.69~2.72)	0.373
≥3.841	2.03 (1.06~3.9)	0.033	2.16 (1.11~4.18)	0.023	2.73 (1.25~5.98)	0.012
P for Trend	0.032	0.022	0.013

Crude model: no other covariates were adjusted. Minimally adjusted model: we adjusted age and sex. Fully adjusted model: we adjusted age, sex, smoking status, hypertension, coronary heart disease, cerebrovascular disease, mean arterial pressure, fasting glucose level and glycosylated hemoglobin.

### Subgroup analyses

**Figure 3:**
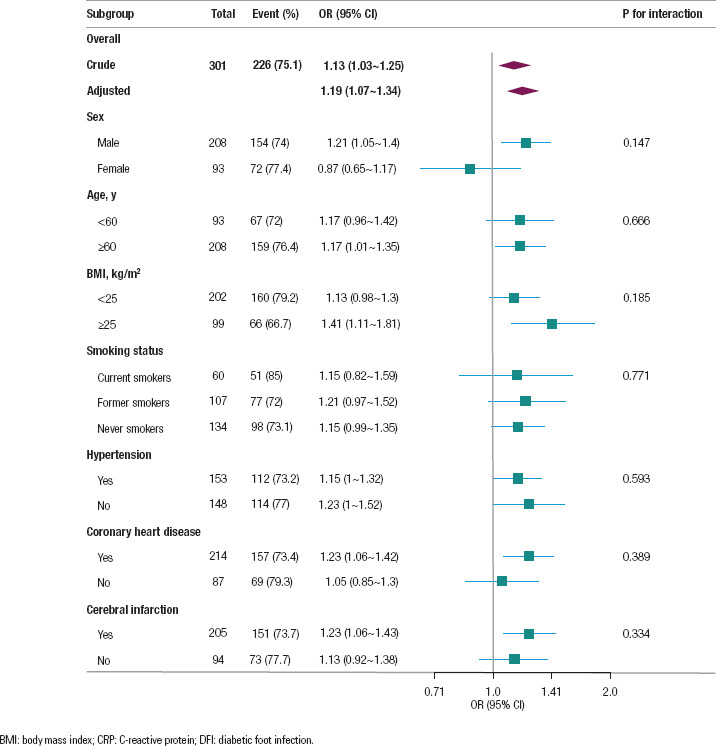
Stratified analyses of the association between CRP/albumin ratio and DFI amputation risk according to baseline characteristics. Except for the stratification factor itself, the stratifications were adjusted for all variables, including age, sex, smoking status, hypertension, coronary heart disease, cerebrovascular disease, mean arterial pressure, fasting glucose level, and glycosylated hemoglobin.

To explore the potential effect of the CRP/albumin ratio more accurately, a stratified analysis was performed to evaluate its relationship with amputation rate (Figure 3). The CRP/albumin ratio was a risk factor in male patients, those aged ≥ 60 years old, and those with a BMI ≥ 25 kg/m^2^, coronary heart disease, or cerebral infarction. No significant interaction was observed between the subgroups (P > 0.05).

### ROC analysis

**Figure 4:**
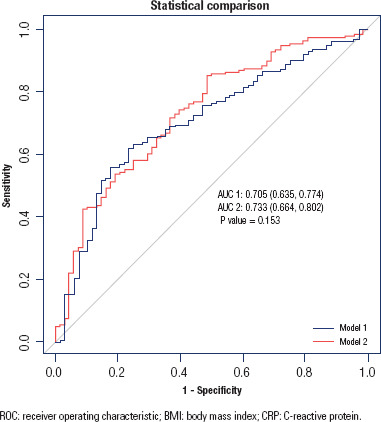
ROC curves evaluated the predict value of baseline risk model and fitting model. Model 1 is the baseline risk model including age, sex, smoking status, BMI, diabetes duration, hypertension, coronary heart disease, cerebrovascular disease, mean arterial pressure, fasting glucose level, and glycosylated hemoglobin and model 2 is the fitting model of the baseline risk model and CRP/albumin ratio.

ROC curves were generated to evaluate the baseline risk model consisting of age, sex, smoking status, BMI, diabetes duration, hypertension, coronary heart disease, cerebrovascular disease, MAP, fasting glucose level, HbA1c, and the fitting model of the baseline risk model and CRP/albumin ratio. The results demonstrated that the fitting model slightly increased the area under the ROC curve from 0.705 (95% CI: 0.635, 0.774) to 0.733 (95% CI: 0.664, 0.802), although the difference was not statistically significant (P = 0.153) (Figure 4).

## DISCUSSION

DFI-associated amputation is a major cause of mortality in patients with diabetes (^31^). In the present study, the CRP/albumin ratio was significantly and positively correlated with amputation rate. This relationship persisted after adjusting for potential confounders and was more pronounced in patients with severe infections. The association between the CRP/albumin ratio and DFI-associated amputation remained stable in subgroup analyses, suggesting that the CRP/albumin ratio is an independent prognostic predictor in patients with DFI.

A large Chinese multicenter cohort study conducted by Jiang and cols. indicated that the overall amputation rate in individuals with DFI was 19.03% (major and minor amputation rates: 2.14% and 16.88%, respectively) (^32^). Between 2017 and 2019, the average annual rough amputation prevalence of non-traumatic major and minor amputations in individuals with diabetes was 41.5/100,000 and 86.9/100,000, respectively, in Zwolle, the Netherlands (^33^). In Romania, the overall incidence of diabetes in patients undergoing non-traumatic amputation increased from 80.61/100,000 in 2015 to 98.15/100,000 in 2019 (^34^). In Africa, the lower limb amputation rate in individuals with diabetes is 1.9% (^35^). Few studies have been conducted on amputation due to infected DFUs in China, particularly in patients with severe infections. In the present study, the amputation rate was 75.08% (226/301), which was significantly higher than rates reported in previous studies. We believe this is because the included populations had DFI with Wagner grades ≥ 3, as an increment of 1 point in the Wagner ulcer classification criteria corresponds to a 65% increase in the risk of amputation (^36^).

Several studies have been conducted to identify risk factors associated with amputation in patients with DFI. Zhu and cols. identified five independent risk factors for DFU-related amputation: peripheral arterial disease, ulcer site, ulcer severity, neutrophil-to-lymphocyte ratio (NLR), and nutritional status (^37^). Zhang and cols. demonstrated that the WBC count, NLR, CRP level, and Wagner grade were independent risk factors for amputation (^38^). Aragón-Sánchez and cols. showed that skin necrosis, low serum albumin levels, high erythrocyte sedimentation rate (ESR), and high NLR were significantly associated with a higher rate of amputation and recurrence, longer duration of antibiotic therapy, and prolonged hospitalization (^39^). Guo and cols. observed that higher HbA1c levels, lower triglyceride levels, and higher Wagner grades were independent risk factors for lower limb amputation in patients with DFU in central and southern China (^29^).

CRP is a marker of systemic inflammation and severe infection. One study of individuals with DFU and osteomyelitis found that ESR, WBC count, CRP level, and the CRP/albumin ratio were significantly elevated, whereas the albumin level was drastically decreased in patients with osteomyelitis compared to those without osteomyelitis (^40^). In a prospective study, Das and cols. (^41^) demonstrated that an albumin level < 3.0 g/dL and CRP level > 5.0 mg/dL were independent predictors of impaired wound healing after an initial successful endovascular treatment. Another retrospective study in Turkey showed that elevated serum CRP levels and older age were reliable prognostic indicators in patients with DFU (^42^).

Decreased albumin levels in patients with DFU are usually indicative of malnutrition and delayed wound healing. Meanwhile, inflammation lowers albumin levels (^29^). A low serum albumin level is indicative not only of a patient’s state of nutrition but also of the severity of the patient’s condition. A study on the relationship between dietary intake of essential nutrients and inflammation in individuals with DFI showed that high-quality diets are beneficial for decreasing inflammation (^43^). Multiple studies have shown that hypoalbuminemia may be a risk factor for amputation (^39^,^44^-^46^) which is consistent with our findings. Exudation of DFUs, frequent dressing changes, and surgeries lead to a large loss of albumin. These factors further weaken the patient’s immunity, and pathogenic microorganisms can easily invade. Therefore, albumin levels should be dynamically monitored in clinical practice, and timely replenishment of albumin is important for improving disease prognosis in patients with DFI.

The CRP/albumin ratio is a potential predictive marker of several inflammation-related diseases (^12^-^26^). Consistent with Karaca’s study (^27^), we also found that the CRP/albumin ratio was a predictive factor for amputation risk in patients with DFI. We also observed that patients with severe infections who underwent major amputation had higher CRP/albumin ratios. Therefore, CRP and albumin levels should be assessed immediately after admission of patients with severe DFIs, and the CRP/albumin ratio should be calculated. However, the only laboratory indicator included in the Infectious Diseases Society of America and IWGDF (^8^) guidelines for diagnosing the severity of DFIs is a WBC count of > 12,000 or < 4000 cells/mm^3^. In patients with high CRP/albumin ratios, clinicians should pay greater attention to avoiding or minimizing the occurrence of amputations whenever feasible.

CRP and albumin tests are readily accessible in clinical practice and important in primary care hospitals. The CRP/albumin ratio reflects both the degree of infection and the nutritional status of patients with DFI. Therefore, based on the DFI guideline indicators, clinicians should consider the CRP/albumin ratio in the initial diagnosis of patients with DFI to offer a more comprehensive understanding of the severity of DFI and the nutritional status of the patient. Moreover, more detailed treatment plans for patients should be formulated to reduce the length of stay, hospitalization costs, and amputation rates.

This study had some limitations that must be noted. First, DFI is a dynamic pathological process; therefore, dynamic monitoring of infective indicators is essential for infection control. We collected the data at the time of admission; therefore, the results of this study reflect the extent of DFI only at the time of admission. Second, owing to the observational research design, causality could not be deduced from the results. Third, as in all observational studies, uncontrolled potential confounders may exist. Additionally, owing to the limitations of the database, we had no information on other nutritional indicators. This should be considered carefully in future real-world studies. Future studies should include randomized controlled trials to confirm the causal relationship between the CRP/albumin ratio and amputation.

In conclusion, the CRP/albumin ratio is a potential predictive factor for amputation in patients with DFIs. Consequently, physicians should pay close attention to screening and initiate timely interventions to improve patient outcomes and avoid or minimize amputation.
