# Supplementary material for: Higher C-reactive protein/albumin ratio is a potential marker for predicting amputation in patients with diabetic foot infection
Source: Arch Endocrinol Metab. 2025 Jun 27;69(3):e240397. doi: 10.20945/2359-4292-2024-0397 (PMC12403742; doi:10.20945/2359-4292-2024-0397)
Supplement: SUPPLEMENTARY MATERIAL [file 2359-4292-aem-69-03-e240397-Supplementary_Material.pdf]

Table S1. Comparison of general and biochemical characteristics between severe infection group and mild and moderate infection group

Variables	Total (n = 301)	Mild and moderate infection (n = 235)	Severe infection (n = 66)	p
Sex, n (%)				0.185
Male	208 (69.1)	158 (67.2)	50 (75.8)	
Female	93 (30.9)	77 (32.8)	16 (24.2)	
Age, years, mean ± SD	65.2 ± 12.0	65.7 ± 12.0	63.7 ± 11.8	0.229
BMI, kg/m^2, mean ± SD	23.2 ± 3.5	23.2 ± 3.5	22.9 ± 3.5	0.553
Mean arterial pressure, mmHg, mean ± SD	59.0 ± 16.9	58.8 ± 16.8	59.6 ± 17.3	0.738
Smoking status, n (%)				0.239
Current	60 (19.9)	42 (17.9)	18 (27.3)	
Never	107 (35.5)	86 (36.6)	21 (31.8)	
Former	134 (44.5)	107 (45.5)	27 (40.9)	
Duration of diabetes, years, mean ± SD	15.4 ± 9.5	15.3 ± 9.4	15.5 ± 9.7	0.9
DFI microbial community, n (%)				0.84
Gram-positive bacterial	100 (34.2)	76 (33.5)	24 (36.9)	
Gram-negative bacterial	74 (25.3)	59 (26)	15 (23.1)	
Mixed infection	118 (40.4)	92 (40.5)	26 (40)	
Hypertension, n (%)				0.686
Yes	153 (50.8)	118 (50.2)	35 (53)	
No	148 (49.2)	117 (49.8)	31 (47)	
CAD, n (%)				0.13
Yes	214 (71.1)	172 (73.2)	42 (63.6)	
No	87 (28.9)	63 (26.8)	24 (36.4)	
Cerebrovascular disease, n (%)				0.665
Yes	205 (68.6)	159 (67.9)	46 (70.8)	
No	94 (31.4)	75 (32.1)	19 (29.2)	
Fasting glucose, mmol/L, mean ± SD	10.4 ± 4.9	9.9 ± 4.6	12.2 ± 5.5	0.001
HbA1c, %, mean ± SD	9.0 ± 2.3	8.9 ± 2.3	9.3 ± 2.2	0.205
RBC, ×10^{12}/L, mean ± SD	3.8 ± 0.7	3.8 ± 0.7	3.7 ± 0.7	0.253
Hemoglobin, g/L, mean ± SD	112.8 ± 22.7	113.9 ± 22.5	109.0 ± 23.4	0.129
WBC, ×10^9/L, mean ± SD	11.6 ± 5.8	10.7 ± 5.5	14.8 ± 5.5	<0.001
Neutrophils, %, mean ± SD	78.5 ± 10.4	76.7 ± 10.5	85.1 ± 6.7	<0.001
Platelet, ×10^9/L, mean ± SD	296.4 ± 115.9	293.2 ± 113.3	307.9 ± 124.7	0.361
CRP, mg/L, mean ± SD	92.7 ± 78.1	75.1 ± 69.0	155.3 ± 77.0	<0.001
Albumin, g/L, mean ± SD	30.8 ± 6.0	31.6 ± 5.7	27.6 ± 5.8	<0.001
CRP/albumin, median (IQR)	2.4 (0.8, 4.7)	1.8 (0.6, 3.6)	4.7 (3.4, 7.5)	<0.001

Note – Abbreviations: BMI, body mass index; CAD, coronary heart disease; CRP, C-reactive protein; DFI, diabetic foot infections; HbA1c, glycosylated hemoglobin; IQR, interquartile range; RBC, red blood cell counts; SD, standard deviation; WBC, white blood cell count.

Copyright© AE&M all rights reserved.
